# Low-Cost FPGA-Based Electronic Control Unit for Vehicle Control Systems

**DOI:** 10.3390/s19081834

**Published:** 2019-04-17

**Authors:** Javier Pérez Fernández, Manuel Alcázar Vargas, Juan M. Velasco García, Juan A. Cabrera Carrillo, Juan J. Castillo Aguilar

**Affiliations:** Department of Mechanical Engineering, University of Málaga, 29071 Málaga, Spain; javierperez@uma.es (J.P.F.); manuel.alcazar@uma.es (M.A.V.); juanmav@uma.es (J.M.V.G.); jcabrera@uma.es (J.A.C.C.)

**Keywords:** data acquisition, ECU, FPGA, TCS, vehicle control

## Abstract

The development of new control algorithms in vehicles requires high economic resources, mainly due to the use of generic real-time instrumentation and control systems. In this work, we proposed a low-cost electronic control unit (ECU) that could be used for both development and implementation. The proposed electronic system used a hybrid system on chip (SoC) between a field-programmable gate array (FPGA) and an Advanced RISC (reduced instruction set computer) Machine (ARM) processor that allowed the execution of parallel tasks, fulfilling the real-time requirements that vehicle controls demand. Another feature of the proposed electronic system was the recording of measured data, allowing the performance of the implemented algorithm to be evaluated. All this was achieved by using modular programming that, without the need for a real-time operating system, executed the different tasks to be performed, exploiting the parallelism offered by the FPGA as well as the dual core of the ARM processor. This methodology facilitates the transition between the designing, testing, and implementation stages in the vehicle. In addition, our system is programmed with a single binary file that integrates the code of all processors as well as the hardware description of the FPGA, which speeds up the updating process. In order to validate and demonstrate the performance of the proposed electronic system as a tool for the development and implementation of control algorithms in vehicles, a series of tests was carried out on a test bench. Different traction control system (TCS) algorithms were implemented and the results were compared.

## 1. Introduction

Designing, implementing, and validating active safety systems in vehicles require a high need for resources and materials [1]. In the implementation and validation stages, it is necessary to use a real vehicle or part of one in the form of a test bench. This process is required to validate the results provided by the simulations prior to their final installation in the vehicles.

In order to minimize the costs and the risk involved in carrying out tests with real vehicles on the road, an attempt is made to leave those vehicles for the final implementation stage. Therefore, the validation and part of the implementation are performed on a controlled test bench using hardware-in-the-loop (HIL), where components of the real vehicle are integrated into the simulation. In this transition between the test bench and the vehicle, electronic systems are typically changed to less expensive ones, which makes it difficult for the system to behave as the one developed on the bench.

Electronic control units (ECUs) incorporated in test benches are based on hybrid systems between a processor with a real-time operating system and a field-programmable gate array (FPGA) in charge of acquiring and processing data from the sensors [2]. These systems tend to be of limited production, which makes them more expensive.

Furthermore, they usually require independent input/output modules for the different controlled signals. After the development, the algorithm is transferred to the vehicle integrating the control software in a microcontroller or in an application-specific integrated circuit (ASIC), reducing the cost of the control unit installed in the vehicle. Electronic control units in commercial vehicles are often hidden and limited information about their internal architecture is available. Even less information is found about the software and how the different control systems are integrated. This is a barrier that this article tries to overcome, offering open hardware electronics that can be replicated by other researchers.

Currently, these hybrid systems, which combine a processor and an FPGA [3,4], are reducing their cost. As a consequence, it is viable to incorporate them into the final product. Thanks to that possibility, the electronic system can be the same during both the validation and the implementation stages. If we compare these electronics with an ECU having an ASIC, the FPGA implies an additional cost, but the ASIC has a fixed hardware defined in the design stage of the vehicle. Therefore, electronics with this flexibility must be compared with control electronics for research purposes. These, in comparison with our proposal, have higher prices, leading to the use of the term “low-cost”.

This system reduces development time and extends the power of the control algorithm, since it can use the resources offered by an FPGA at a low cost. One of the main advantages of the hybrid system is its capacity of modular programming. It allows the implementation of vehicle safety algorithms without the need of a real-time operating system. The main advantage of this contribution is that enables an easier and faster way of incorporating control algorithms from different sources. 

In hybrid systems, different methods are used to program the main processor: block-based language [5], programming of a real-time operating system [6], and direct coding in low-level language such as C or C++ [7]. The first option, based on blocks, is associated with the software property of a private company. Despite adding an important cost to the development, it is the option with the least technical complexity. The second is based on a real-time operating system that ensures that all implemented tasks comply with their respective deadlines and thus ensures real time. This option requires a technical complexity that is far superior to others. As a consequence, a great level of knowledge in real-time system programming is required. Finally, the option of using low-level language (C or C++) directly as the single program in the processor presents coordination problems between the different tasks. Therefore, programmers prefer to use a real-time system that executes tasks in parallel. The execution of the operating system itself limits the computing capacity of the processor. For this reason, less complex algorithms are required. However, implementing a low-level code with a single task allows the processor to execute complex algorithms, optimizing computational capabilities. This work proposes to take advantage of the parallelism offered by the FPGA with the possibility of integrating a soft processor that executes a C or C++ code to execute low load and repetitive tasks [8] while freeing the processor for the execution of the algorithm without the operating system. The ECU uses the Zynq-7000 chip manufactured by Xilinx which also includes a dual-core Advanced RISC (reduced instruction set computer) Machine (ARM) processor [9]. This also allows the execution of two programs in parallel at a high-clock rate, which makes it perfect for the main control loop and data recording. On the other hand, the soft processor integrated in the FPGA is responsible for the acquisition and pre-processing of data in parallel. The communication between the processors is carried by means of a shared memory through the advanced extensible interface (AXI) bus and a first-in, first-out (FIFO) memory [10,11].

The proposed ECU was developed to be used in vehicles [12,13,14]. The performance of the proposed ECU was evaluated on a test bench [15] using parts of the actual vehicle [16]. As an example, a traction control system (TCS) was implemented on the drivetrain of a 40 kW electric motorcycle. Different controllers were tested using a mathematical model of the system. After the implementation, a comparison was given to demonstrate and validate the capabilities of the ECU.

This article begins with the description of the test bench and the electronic system in Section 2. The modular programming is introduced in Section 3. The mathematical model of the controller is described in Section 4. The performance of the whole system is verified through tests included in Section 5. Conclusions are drawn in Section 6.

## 2. Test Bench and Electronics

To evaluate the ECU, the drive unit of an electric vehicle was required. This system included an electric motor, a transmission, a swingarm, a wheel, and a device that simulated contact with the asphalt. All this allowed us to simulate the real speed of the vehicle. The control of the vehicle was carried out by a low-cost electronic-based FPGA and ARM processor, which represented the main contribution of our work.

### 2.1. Test Bench

**Figure 1:**
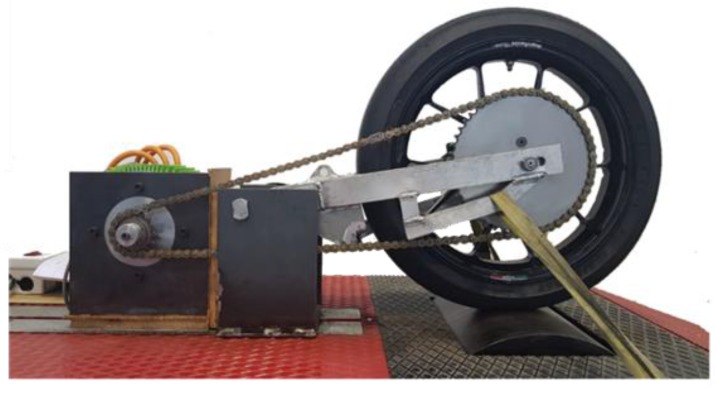
Rear drivetrain setup in the test bench.

A test bench was used to evaluate the performance of the proposed ECU (Figure 1). A roller with high inertia [17,18] was used to simulate the asphalt. The vertical load of the tire was modified by an external force applied to the swingarm to reproduce changes in grip conditions. It included an inertia roller (I_roller_ = 6.5 kg·m^2^) and an inductive sensor with 74 teeth to measure the speed of the roller (R_roller_ = 450 mm). The electric traction system consisted of a permanent magnet synchronous motor (PMSM) model MS1718 from the manufacturer Motenergy (Hartford, WI, USA) with a peak power of 40 kW, a torque of 120 Nm, and an inertia (I_motor_ = 0.096 kg·m^2^). The transmission between the engine and the wheel was carried out by means of a 15-tooth driver sprocket (Z_motor_ = 15) and a 51-tooth driven sprocket (Z_wheel_ = 51). The wheel consisted of an OZ rim and a Dunlop tire 115/70 R17 (R_wheel_ = 318.4 mm) with a total inertia (I_rim_ + I_tire_ = I_wheel_) of 0.18 kg·m^2^.

**Figure 2:**
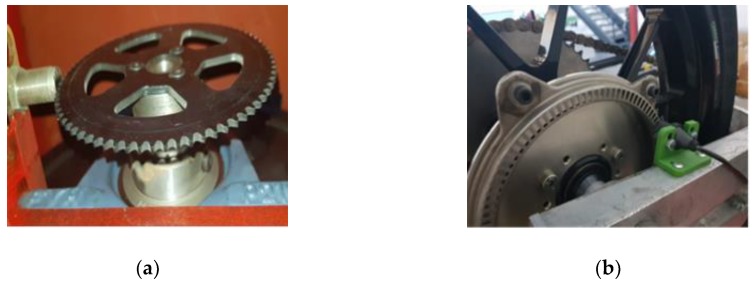
Angular speed sensors mounted on the test bench. (**a**) Roller angular speed and (**b**) wheel angular speed.

In order to carry out the control, the speed of the driving wheel and the vehicle had to be measured. The speed of the vehicle was obtained from the inductive encoder that was part of the test bench (Figure 2a). On the other hand, the speed of the driving wheel was obtained through a controller area network (CAN) communication offered by the power inverter that controlled the engine. These data were delayed due to the fact that the communication and the measurement of speed were indirect, differentiating the position obtained by the absolute encoder that the motor used. Therefore, a hall effect sensor with a 100-tooth plate (Figure 2b), similar to those used in commercial motorcycles, was installed. Thus, the developed controller uses low-cost sensors that are easy to implement in a real vehicle.

The transformation between the square wave signal coming from the sensors requires a high frequency processing in order to not lose any change in the signal [19]. This was implemented directly in the FPGA with the help of a high frequency clock, which allowed counting the time between pulses and filtering the signal within the execution time of the main control loop. Therefore, there was no interruption of the main control loop, which ensured the compliance with the established deadlines.

### 2.2. Electronic Control Unit (ECU)

In order to design and implement an ECU in a vehicle, the following requirements must be met:

- Low cost
- Real-time execution (≤1 ms control loop)
- CAN communication to other ECUs
- Digital and analog I/O
- Fast data logger for multiple channels (≥100 Hz) (20 channels)
- Host communication

Both the datalogger system and the host communication would, in principle, only be strictly necessary in a test vehicle, although the reality is that both systems are currently included in production vehicles. 

**Figure 3:**
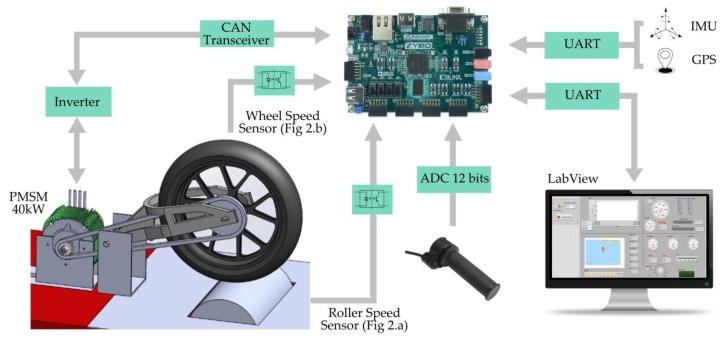
Connection diagram of the system components.

The main reason is the connectivity of the vehicle with the user through a web or mobile app that enables them to know the state of the vehicle (i.e., state of battery charge). The datalogger also allows the manufacturer to know the reason for breakdowns and predict future failures based on the data collected. Therefore, we proposed to use a system on chip (SoC) that integrated an ARM processor and an FPGA whose programming is discussed in Section 3. Next, the components that are part of the system are described, as well as the communication system used between them (Figure 3).

#### 2.2.1. Inverter

Power delivery was managed through the CAN communication established between the power inverter and our device. This required a transceiver that adapted the digital levels and isolated communication with the high voltage system (HVS). This communication gave access to the variables controlled by the inverter such as motor speed, stator current, and throttle input.

#### 2.2.2. Speed Sensors

As previously mentioned, the acquired signal was digital. This signal had an amplitude of 12 V. It was adapted to 3.3 V using a high frequency optocoupler with a Schmitt trigger to reduce electrical noise in the acquired signal. The frequency of this signal was translated into angular velocity according to the number of teeth per turn that the phonic wheels had.

#### 2.2.3. Throttle

The torque target (CAN power inverter input) was set according to the position of the throttle grip and the output of the control algorithm. The position of the throttle grip was set by the user through a potentiometer with a 12-bit analog-to-digital converter. This one communicated through a serial peripheral interface (SPI) with the FPGA, coding the voltage between 0 and 5 V.

#### 2.2.4. PC Host

The electronic control system was connected to a PC via UART communication in order to speed up the parameter adjustment process and to visualize the test in real time. The system sent data packets at low frequency (≅100 ms). Meanwhile, the host ran a LabView® application that processed the data packets by displaying them on a screen in different graphs.

#### 2.2.5. Additional Data

For real road tests, the recording of acceleration data with an inertial measurement unit (IMU), as well as the position and speed of the global positioning system (GPS), is important in order to replicate the tests and to compare and establish the efficiency of the results. In commercial vehicles, both sensors add extra value by providing the user with information about their driving patterns. Therefore, although they are not necessary for bench testing, the control electronics include two soft processors that perform data fusion and GPS calculations in parallel without affecting the main control loop.

## 3. Programming

**Figure 4:**
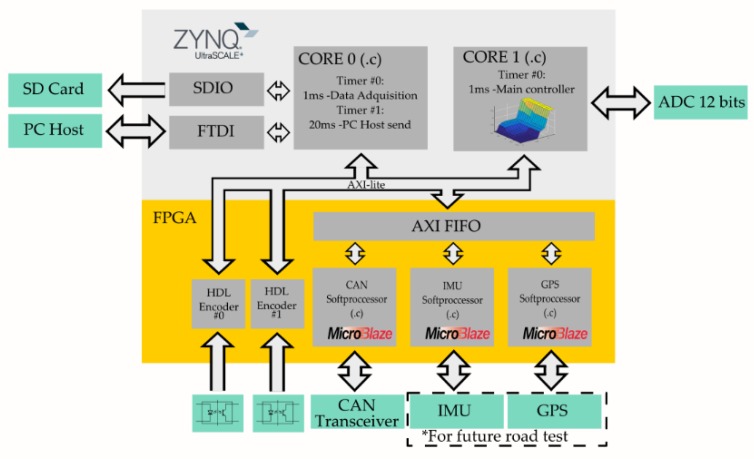
System-on-chip (SoC) Zynq-7000 hardware design describing inter-modular communication, as well as the code that executes or describes each functionality.

**Figure A1:**
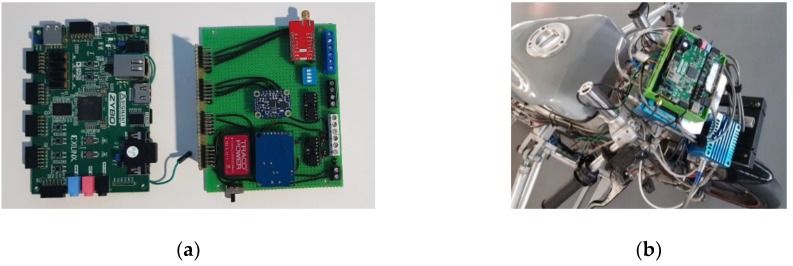
(**a**) Electronic control unit and (**b**) implementation in a two-wheeled vehicle.

The programming of the Zynq-7000 device was the main objective of this work, providing a modularity that offers the advantage of executing different low-level codes in parallel and avoiding conflicts of access to memory or peripherals. There are three main resources in this architecture: two ARM cores and an FPGA (Figure 4). In our case, the distribution was made taking into account the resources that each task required. Thus, the tasks related to acquisition and data fusion were implemented in the FPGA, since the reading of data is direct and does not require an intermediate memory. On the other hand, data recording and control require the use of a flash memory and more computational capacity. Therefore, one of the ARM cores was dedicated to running the control algorithms, which required a high computational cost, whereas the other core was used for recording purposes since it had direct access to the SD memory. For the design of the prototype, a low-cost ZYBO development board from Digilent (Pullman, WA, USA) was used for the design of the prototype (see prototype photograph in Appendix A, Figure A1). It included an SoC XC7Z010-1CLG400C (Xilinx, San Jose, CA, USA), whose programming is described in Figure 4.

### 3.1. Dual-Core ARM

The main processor was devoted to performing tasks that required greater computational capacity. These tasks were data recording and execution of the main control loop. Data recording required encoding the data packets of the different inputs and outputs and writing to an external memory. The main control loop had to combine the inputs obtained through an algorithm that calculated the controlled signal.

In order to coordinate both processors in parallel, it was necessary to establish the memory zones where each core worked so that there were no resource conflicts and both programs were executed correctly. On the other hand, it was necessary for all the codes to be synchronized with the same interruption. To do so, a highly flexible FPGA was used. A 32-bit timer was generated that created an interruption in all processes. In the case of the ARM processor, independent private interruptions were used for each processor so that both cores were interrupted at the same time.

#### 3.1.1. Core 0

This core was used to record high-frequency data and send low-frequency packets to the host. The data recording was done on a MicroSD card for easy extraction and transfer of information to any other device. The data of the inputs and outputs made up a 256-byte packet that was transferred to the SD card memory by means of a double circular buffer of 128 elements. This optimized data recording on the SD card meeting the time requirements of 10 ms for a recording at 100 Hz. The packet was sent to the host via UART communication and the value of the parameters configured on the host was updated. 

This processor was also used in the initialization process to establish the synchronization frequency of the system by configuring and initializing the global timer.

#### 3.1.2. Core 

This core was intended for the execution of the control algorithm. Thus, the algorithm had access to a high-frequency processor and to the measured variables. In this case, the throttle through SPI communicated with a 12-bit analog-to-digital converter (ADC). The code of this processor was modified to establish different control logics such as the on/off controller (relay), proportional-integral-derivative (PID) controller, or fuzzy logic with a loop time of 1 ms (1 kHz). Thanks to the modular configuration, only one core was assigned to the execution of the algorithm, thus allowing the programming of complex algorithms that required higher computational cost.

### 3.2. FPGA

The use of an FPGA provided great flexibility to the system. Repetitive processes of low computational cost and the conditioning of signals that required asynchronous temporary interruptions could be implemented, as is the case of digital speed signals. When working with the FPGA, two types of codes must be generated and stored in the non-volatile memory that is loaded when the system is started. The first codes include the hardware description of the elements integrated in the FPGA with logical blocks, such as the reading of the encoder in hardware description language (HDL), and the hardware description of the soft processors provided by the manufacturer Xilinx with the microBlaze processor. The second codes to be generated are the low-level programs (C or C++) that are executed in each of the soft processors. Both types of codes are compiled and loaded simultaneously with the main processor programs into the non-volatile memory.

#### 3.2.1. HDL Encoder

A hardware description language (HDL) physically implements the acquisition of signals from encoders that codify the velocity value in the period of the generated digital signal. A binary counter with a 40 MHz high frequency clock was used to measure the period of the signal. This counter was reset each time a rising edge was detected. Both edges were not used to increase the accuracy since the detection of the tooth/hole or absence of it may not have been symmetrical. The value of the period obtained in each rising edge was stored in a circular buffer that saved the values of the last periods. A mean filter was used to reduce signal noise. This preprocessor filtered the data during a time shorter than the execution time of the main control loop to avoid delays in the measurement.

#### 3.2.2. CAN Soft Processor

Processing CAN signals and decoding them have a low computational cost, but require a constant use of resources. This makes a soft processor ideal, since it can be implemented in the FPGA in parallel and relieves the main processor of a repetitive task. The main task was to decode the received messages by collecting the identifiers and applying the different scales to each variable. Finally, the decoded variables were stored in a FIFO memory so that the main processors could have access to them. In addition, this processor was in charge of sending the orders coming from the control algorithm—in this case, the percentage of throttle required by the inverter.

#### 3.2.3. IMU Soft Processor and GPS Soft Processor (Future Road Test)

The data fusion between the accelerations obtained from an IMU and the GPS data requires pre-processing to reduce the error of the measured variables. Therefore, the use of a soft processor met the characteristics of this type of calculation. Therefore, as with the decoding of CAN messages, the main processor did not have to perform repetitive tasks that consumed its resources. The calculated variables were stored in a memory where the main processors had access.

## 4. Controller

The power delivery management through the optimization of the slip has been used in vehicles since the appearance of the Bosch TCS [20]. Several approaches can be used to perform the control of a wheel during traction. Among them, we can find two states [21], PID [22], and fuzzy-logic controllers [23,24]. There are also other approaches that combine PID with fuzzy logic [25,26,27]. The design of an optimized controller capable of managing power delivery during traction is beyond the scope of this work. In this paper, three of the most widely used controllers were programmed and implemented to demonstrate the adaptability and performance of the proposed ECU. The mathematical model was simulated using Simulink®. This model allowed fitting the parameters of the three controllers.

### 4.1. Test Bench Math Model

The following mathematical equations model the behavior of the wheel and the test bench: wheel dynamics, Equations (1)–(3); roller dynamics, Equation (4); and the contact between both, Equations (5) and (6):

(1) $$I_{\mathrm{wheel}}^{\prime }{\dot{\omega }}_{\text{wheel}}=T_{\mathrm{motor}}\tau \;-\;F_{x}(\kappa ,F_{z})R_{\mathrm{wheel}}{\;-c\;\omega }_{\mathrm{wheel},}$$

(2) $$I_{\mathrm{wheel}}^{\prime }{\;=\;I}_{\mathrm{wheel}}^{\;}{\;+\;I}_{\mathrm{motor}}^{\;}\tau^{2},$$

(3) $$\tau \;=\;\frac{Z_{\mathrm{wheel}}}{Z_{\mathrm{motor}}}\;,$$

**Figure 5:**
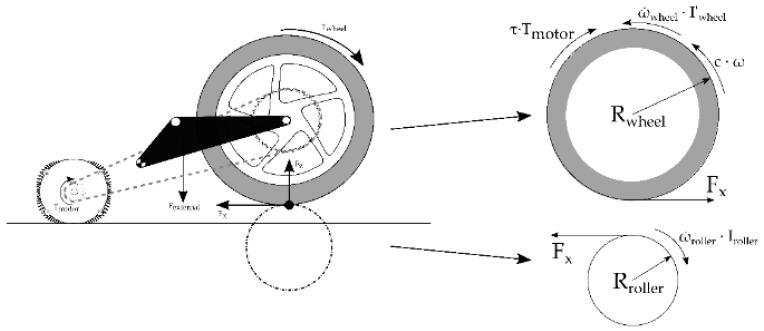
Free body diagram of the wheel and test bench roller.

The equations that model the wheel and the roller are obtained from the dynamic equilibrium, both being decoupled from each other (Figure 5). The contact force (F_x_) relates both speeds through the longitudinal slip (κ) between the wheel and the roller. This friction is modelled using Pacejcka’s magic formula [28] in its pure longitudinal model. The parameters of the motorcycle tire are obtained from Sharp et al. [29], which provides the data of a 160/70 R17 tire with similar characteristics.

(4) $$I_{roller}{\dot{\omega }}_{roller}{=\;F}_{x}{(\kappa ,F}_{z})R_{roller}$$

(5) $$F_{x}\left(\kappa ,F_{z}\right)\;=\;D_{x}{\sin[C}_{x}{\arctan\{B}_{x}(F_{z})\kappa \;-\;E_{x}{(B}_{x}s-\arctan\left(B_{x}(F_{z})\;\kappa \right))\}]$$

(6) $$\kappa \;=\;-\frac{\omega_{roller}R_{roller}{\;-\;\omega }_{wheel}R_{wheel}}{\omega_{roller}R_{roller}}$$

The parameters that model the system (Table 1) are obtained from different data sources: the geometric parameters (R_wheel_, R_roller_, Z_wheel_, and Z_motor_) are measured directly, whereas the moments of inertia (tire, rim, motor, and roller) are provided by the different manufacturers. The only parameter that requires empirical testing is the damping coefficient that models transmission and bearing losses. A value of 4.64 Nms/rad (Equation (8)) is obtained by knowing the deceleration of the system without being in contact with the roller Equation (7):

(7) $$I_{wheel}^{\prime }{\dot{\omega }}_{wheel}{=\;T}_{moter}{\tau \;-\;F}_{x}{(\kappa ,F}_{z}{)R}_{wheel}{\;-\;c\;\omega }_{\text{wheel}},\;\{\begin{array}{ccc} T_{motor} & =\;0 & \left(Throttle\;=\;0\right)\\ F_{x} & =\;0 & \left(Raised\;Wheel\right) \end{array}$$

(8) $$c\;=\;\frac{I_{w\;}^{\prime }{\dot{\omega }}_{wheel}}{\omega_{wheel}}$$

The measurement of the vertical force (F_z_) applied to the tire by the external force of the swingarm is calculated by measuring the deformation of the tire. Finally, to model the electrical system, the motor map provided by the manufacturer is used as well as a model of the power inverter (see [30]). As can be seen, the power inverter output is modelled as a second order system, Equation (9), where s is the throttle percentage;

(9) $$f\left(s\right)\;=\;\frac{1}{0{.007\;s}^{2}\;+\;0.134\;s\;+\;1}\;.$$

### 4.2. Controllers

**Figure 6:**
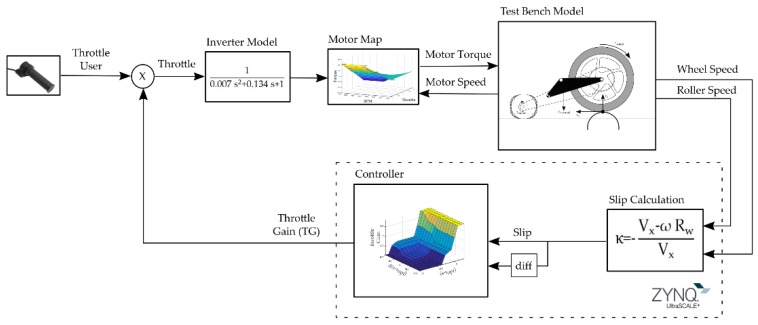
Control system block diagram implemented in the simulation. ECU control algorithm is represented inside the dashed box.

TCSs are designed to prevent loss of traction by controlling the slippage between the driving wheel and the road (in our case the roller). In order to analyze the effectiveness of the proposed control system, a comparison between three TCSs found in the literature is included. These control systems present different levels of complexity, from the implementation of a simple relay system to a fuzzy-logic control. To adjust the parameters, the general scheme implemented in Simulink^®^ is presented (see Figure 6), where the different controllers are simulated and finally implemented in the developed electronic control system.

The code used to control the model after its correct adjustment is compiled in C language and included in the processor in charge of the execution of the real-time control algorithm. Real tests allow the performance of the electronics to be evaluated. In addition, since there is no operative system, real-time performance is assured by design, due to parallelism of the soft processors in the FPGA and dual core ARM processors. Therefore, it is guaranteed that there are no collisions between tasks. On the other hand, it was verified that the execution of each implemented task was accomplished in a shorter time than its deadline, thanks to the debugging software offered by the manufacturer XILINX.

#### 4.2.1. Relay Controller

The relay controller is the simplest since it only uses a slip limit (s_opt_) based on the type of road, in our case constant, to either activate the accelerator or not. This controller uses a hysteresis system to avoid oscillations in the control. Therefore, a working zone around the optimal slip is defined:

(10) $${TG}_{new}\left({\kappa ,TG}_{old},\kappa_{opt}\right)\;=\;\{\begin{array}{ccc} 1 & if & \;\kappa \;<\;0.75\;\kappa_{opt}\\ {TG}_{old} & if & 0.75\;\kappa_{opt}\;\leq \;\kappa \;\leq \;1.25\;\kappa_{opt}\\ 0 & \mathrm{if} & \;\kappa \;\text{>}\;1.25\;\kappa_{opt} \end{array}\;.$$

#### 4.2.2. PID Controller

PID controllers are well known in industry for their versatility and easy implementation to control linear systems. In this case, the throttle is modulated according to the slippage. To do this, the current value of the slip error, Equation (11), its variation in time through an integral term as well as its derivative Equation (12) are used. To adjust this controller, the model is linearized and the parameters are tuned according to Ziegler–Nichols. This controller is not optimal for highly nonlinear systems such as asphalt–tire contact. For this reason, a controller capable of adapting to the non-linearity of the system is proposed below:

(11) $$e_{s}\;=\;\kappa \;-\;\kappa_{opt}\;,$$

(12) $$\mathrm{TG}\left(e_{s}{,K}_{p}{,K}_{i}{,K}_{d}\right){\;=\;K}_{p}e_{s}{+K}_{i}\overset{t}{\underset{0}{\int }}e_{s}{dt\;+\;K}_{d}\frac{d}{dx}e_{s}\;.$$

#### 4.2.3. Fuzzy Logic

**Figure 7:**
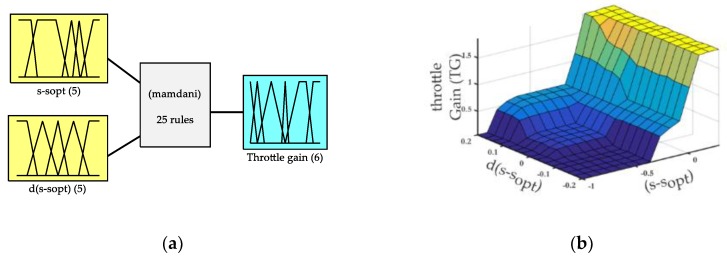
Fuzzy-logic controller: (**a**) membership functions, (**b**) system output surface.

Finally, a controller capable of adapting to the non-linearities presented in the system is simulated. Therefore, a controller based on fuzzy logic, which is widely known for its robustness and widespread use in control systems in vehicles, was programmed. Since the development of this algorithm was not the main objective of this work, a controller previously developed by our research group [30] was used. The Simulink^®^ model optimizes the parameters for the specific task. This controller takes the slip and its derivative as inputs (Figure 7a). Thus, it is able to identify the area in which the tire works and regulate the slip to maximize the distance travelled.

Finally, the rules and functions of membership are integrated in a map (Figure 7b) to simplify the integration of the algorithm into the developed electronic control system.

This map is discretized to generate a look-up table that is stored in the memory of the processor in charge of the control. The access to the data is made by linear interpolation using the error and its derivative as input. The output of the look-up table is the value of the proportional gain to be applied in the power inverter.

## 5. Test and Results

In order to verify the efficiency of the electronic controller, different tests were carried out to evaluate the real-time performance, as well as to create a platform to compare different control algorithms. The electronic system was able to meet the time requirements in all cases.

In this case, the three controllers described in the previous section were programmed. Tests were carried out from standstill to 30–40 m/s with low vertical load on the wheel and maximum acceleration to produce high slips. The results obtained for each controller, with and without TCS, are discussed below.

### 5.1. Controller Off

**Figure 8:**
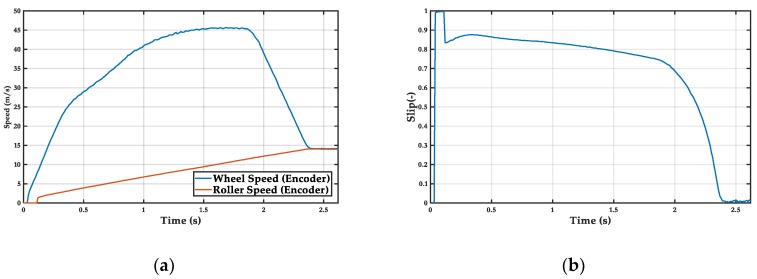
Controller off test: (**a**) wheel and roller speed, (**b**) wheel slip.

**Figure A2:**
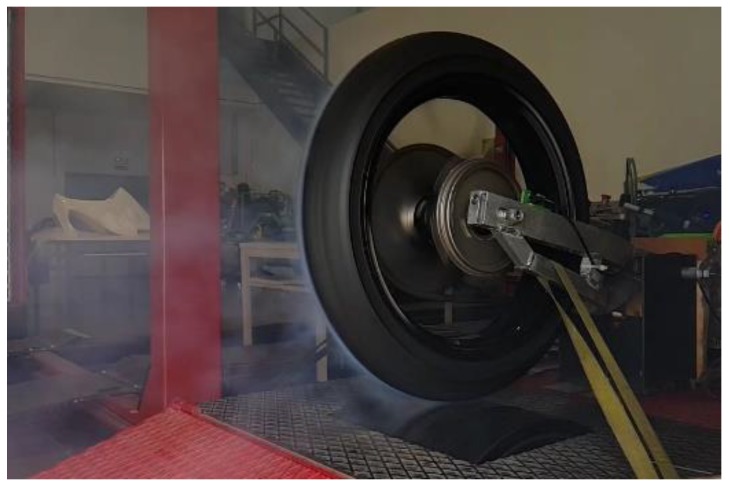
Burnout due to excessive slippage without TCS.

Large slip occurs due to the low load and the high power of the motorcycle, leading to burnout (Figure 8) (see burn out during the tests in Appendix A, Figure A2). This effect has to be avoided in order to increase the life of the tire and to enhance vehicle handling and safety. The following controllers prevent the wheel from skidding while maintaining an optimal slip value.

### 5.2. Relay Controller

**Figure 9:**
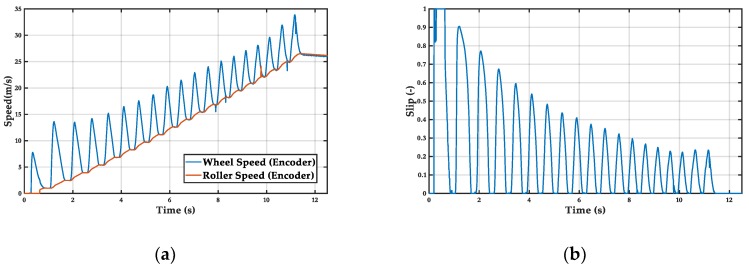
Relay controller: (**a**) wheel and roller speed, (**b**) wheel slip.

The relay controller has high oscillations due to the transition from a stable state with low acceleration to an unstable state with greater slip. This causes the slip to move away from the optimum resulting in lower acceleration. Mean acceleration = 2.5 m/s^2^ (Figure 9).

### 5.3. PID Controller

**Figure 10:**
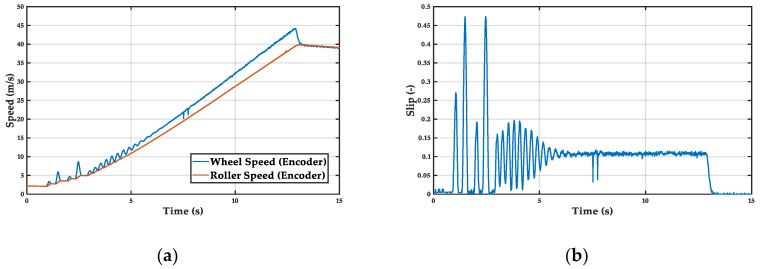
PID controller: (**a**) wheel and roller speed, (**b**) wheel slip.

The PID controller, unlike the previous one, is able to maintain a more constant slip. However, the adjustment of the integral term that allows this behavior makes its response slow at low speed. This is due to the non-linearity of the tire dynamics at very low speed. Mean acceleration = 3.4 m/s^2^ (Figure 10).

### 5.4. Fuzzy Logic

**Figure 11:**
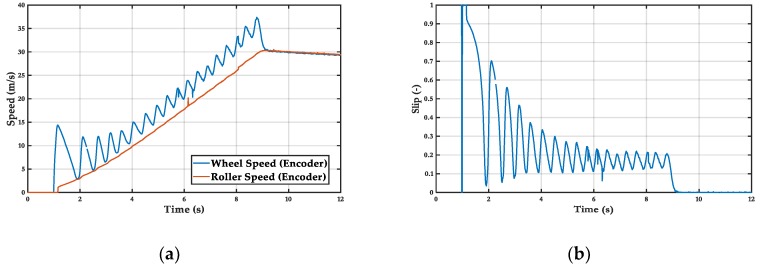
Fuzzy-logic controller: (**a**) wheel and (**b**) roller speed, wheel slip.

Finally, the controller based on fuzzy logic is capable of obtaining greater acceleration, adapting better to the dynamics of the system. On the other hand, it has greater oscillations than the PID, which shows that the fuzzy works in the nonlinear zone, which coincides with the maximum grip and therefore maximizes the travelled distance. Mean acceleration = 4 m/s^2^ (Figure 11).

## 6. Conclusions and Future Work

The development and validation of a real-time ECU was carried out in this work. Thanks to the use of modular programming, all the resources provided by the parallelism of the FPGA as well as by the dual-core processor were exploited. Therefore, the deadlines imposed by the system were met without the need for a complex real-time operating system. Furthermore, control was achieved with low-level language providing a very fast response that allowed the implementation of complex control algorithms from simulation platforms such as Simulink®. This low-cost ECU achieves the characteristics imposed to develop and implement control algorithms in vehicles, enabling the recording of the data obtained from sensors and installed actuators. All this allows us to implement the system directly from hardware-in-the-loop platforms to commercial vehicles, thus saving deployment and development time.

The performance of the proposed electronic system was verified by programming and testing TCS controllers as can be seen in the videos included in this paper Supplementary Material. It was shown that this ECU could be used for the development of the TCS as well as other active safety algorithms in vehicles such as the following: anti-lock braking systems (ABS), electronic stability control (ESC), and collision avoidance systems. 

Modular programming takes advantage of the data resources offered by an SoC such as the Zynq-7000. One of the processor cores was used for the control algorithm while the other core performed data recording. The FPGA performed both the readings of the sensors needed for each controller and the preprocessing, using HDL language as well as soft processors in parallel.

Future works will include evaluating the performance of a wider range of active safety system controllers using test vehicles. In addition, the IMU and GPS allow data fusion techniques that provide information on the state of the vehicle and the road, which can be integrated into the control algorithm, increasing the performance of the ECU developed in this paper.

## Figures and Tables

**Table 1:** Test bench model parameters and variables.

	Parameters		Variables
$R_{\mathrm{wheel}}$	Wheel radius	${\dot{\omega }}_{\mathrm{wheel}}$	Wheel Angular Acceleration
$R_{\mathrm{roller}}$	Roller radius	${\dot{\omega }}_{\mathrm{roller}}$	Roller Angular Acceleration
$Z_{\mathrm{wheel}}$	Wheel sprocket diameter	$\omega_{\mathrm{wheel}}$	Wheel Angular Speed
$Z_{\mathrm{motor}}$	Motor sprocket diameter	$\omega_{\mathrm{roller}}$	Roller Angular Speed
c	Friction Coefficient	κ	Slip ratio
$I_{\mathrm{wheel}}^{\;}$	Moment of inertia of the wheel	τ	Motor–wheel Transmission Ratio
$I_{\mathrm{motor}}^{\;}$	Moment of inertia of the motor	$T_{\mathrm{motor}}$	Torque applied by the motor
$I_{\mathrm{roller}}^{\;}$	Moment of inertia of the roller	$F_{x}$	Longitudinal wheel force
$I_{\mathrm{wheel}}^{\prime }$	Moment of inertia on wheel hub	$F_{z}$	Vertical wheel force
$D_{x}{,C}_{x}{,B}_{x}{,E}_{x}$	Tire Model Parameters [28,29]		

## Supplementary Materials

The following are available online at https://www.mdpi.com/1424-8220/19/8/1834/s1, Video recordings of the three most relevant tests (TCS off, PID, and fuzzy logic) are attached.

## Appendix A

Figure A1a shows an image of the electronic control and data conditioning unit and its implementation in a two-wheeled vehicle is shown in Figure A1b. The ECU is composed of two electronic boards. The first one is the ZYBO development board which includes an SoC XC7Z010-1CLG400C with the ARM and the FPGA. The second board includes all the peripherals needed to conduct the tests. Figure A2 shows an image of the test performed without TCS where the burnout effect is visualized.

## References

[1] Gruyer D., Choi S., Boussard C., D’Andrea-Novel B. From virtual to reality, how to prototype, test and evaluate new ADAS: Application to automatic car parking. Proceedings of the 2014 IEEE Intelligent Vehicles Symposium Proceedings.

[2] Heidrich L., Shyrokau B., Savitski D., Ivanov V., Augsburg K., Wang D. (2013). Hardware-in-the-loop test rig for integrated vehicle control systems. IFAC Proceedings Volumes.

[3] Nicolas-Apruzzese J., Lupon E., Busquets-Monge S., Conesa A., Bordonau J., García-Rojas G. (2018). FPGA-Based Controller for a Permanent-Magnet Synchronous Motor Drive Based on a Four-Level Active-Clamped DC-AC Converter. Energies.

[4] Sánchez-Solano S., Cabrera A.J., Baturone I., Moreno-Velo F.J., Brox M. (2007). FPGA implementation of embedded fuzzy controllers for robotic applications. IEEE Trans. Ind. Electron..

[5] Rosol M., Pilat A., Turnau A. Real-time controller design based on NI Compact-RIO. Proceedings of the International Multiconference on Computer Science and Information Technology.

[6] Ferreira C.Z., Cardoso R., Meza M.E.M., Ávila J.P.J. (2018). Controlling tracking trajectory of a robotic vehicle for inspection of underwater structures. Ocean Eng..

[7] Giftthaler M., Neunert M., Stäuble M., Buchli J. The Control Toolbox—An Open-Source C++ Library for Robotics, Optimal and Model Predictive Control. Proceedings of the 2018 IEEE International Conference on Simulation, Modeling, and Programming for Autonomous Robots (SIMPAR).

[8] Muñoz-Barron B., Morales-Velazquez L., Romero-Troncoso R.J., Rodriguez-Donate C., Trejo-Hernandez M., Benitez-Rangel J.P., Osornio-Rios R.A. (2012). FPGA-Based Multiprocessor System for Injection Molding Control. Sensors.

[9] ElAzab H.-A.I., Swief R.A., Issa H.H., El-Amary N.H., Balbaa A., Temraz H.K. (2018). FPGA eco unit commitment based Gravitational Search Algorithm integrating plug-in electric vehicles. Energies.

[10] Zhou X., Wang Y., Kuang P. Prototype design of a time-of-flight camera based on Xilinx Zynq7000 SoC platform. Proceedings of the 2018 IEEE International Instrumentation and Measurement Technology Conference (I2MTC).

[11] Wirtz S.F., Cunha A.P.A., Labusch M., Marzun G., Barcikowski S., Söffker D. (2018). Development of a low-cost FPGA-based measurement system for real-time processing of acoustic emission data: Proof of concept using control of pulsed laser ablation in liquids. Sensors.

[12] Guo H., Chen H., Xu F., Wang F., Lu G. (2013). Implementation of EKF for vehicle velocities estimation on FPGA. IEEE Trans. Ind. Electron..

[13] Li H., Du Y. Research on the vehicle ESP system in FPGA. Proceedings of the 2010 Chinese Control and Decision Conference, CCDC 2010.

[14] Kucera P., Pistek V. (2017). Testing of the mechatronic robotic system of the differential lock control on a truck. Int. J. Adv. Robot. Syst..

[15] Chu L., Hou Y., Liu M., Li J., Gao Y., Ehsani M. Development of air-ABS-HIL-simulation test bench. Proceedings of the 2007 IEEE Vehicle Power and Propulsion Conference, VPPC 2007.

[16] De Castro R., Araujo R.E., Freitas D. (2013). Wheel slip control of EVs based on sliding mode technique with conditional integrators. IEEE Trans. Ind. Electron..

[17] Hao R.R., Zhao X.M., Xu Z.G. Auto anti-lock braking system bench test results classification model based on neural network. Proceedings of the 2011 International Conference on Electronics, Communications and Control, ICECC 2011.

[18] Juhás M., Seman P., Bodi S. ABS/TCS Simulator. Slovak University of Technology in Bratislava Institute of Information Engineering, Automation, and Mathematics. Proceedings of the 18th International Conference on Process Control Hotel Titris.

[19] Hace A., Curkovic M. (2018). Accurate FPGA-Based Velocity Measurement with an Incremental Encoder by a Fast Generalized. Sensors.

[20] Reif K. (2014). Brakes, Brake Control and Driver Assistance Systems.

[21] Kogut K., Kolek K., Ros M., Mitkowski W., Kacprzyk J., Oprzędkiewicz K., Skruch P. (2017). A new current based slip controller for ABS. Trends in Advanced Intelligent Control, Optimization and Automation.

[22] Cho K., Kim J., Choi S. The Integrated Vehicle Longitudinal Control System for ABS and TCS. Proceedings of the 2012 IEEE International Conference on Control Applications.

[23] Liu Z., Shi Y., Chen H., Zhang X. Modeling and Simulation of Fuzzy Control to Traction Control System of the Four- wheel-drive Vehicle. Proceedings of the 2010 2nd International Conference on Future Computer and Communication.

[24] Li L., Ran X., Wu K., Song J., Han Z. (2015). A novel fuzzy logic correctional algorithm for traction control systems on uneven low-friction road conditions. Veh. Syst. Dyn..

[25] Li L., He L., Kang M., Yu L. (2012). PID plus fuzzy logic method for torque control in traction control system. Int. J. Automot. Technol..

[26] Chu L., Chao L., Ou Y., Lu W. (2012). Hardware-in-the-loop Simulation of Traction Control Algorithm Based on Fuzzy PID. Energy Procedia.

[27] Olivares-mendez M.A., Sanchez-lopez J.L., Jimenez F., Campoy P., Sajadi-alamdari S.A., Voos H. (2016). Vision-Based Steering Control, Speed Assistance and Localization for Inner-City Vehicles. Sensors.

[28] Pacejka H.B. (2012). Tire and Vehicle Dynamics.

[29] Sharp R.S., Evangelou S., Limebeer D.J. (2004). Advances in the Modelling of Motorcycle Dynamics. Multibody Syst. Dyn..

[30] Aguilar J.J.C., Fernández J.P., García J.M.V., Carrillo J.A.C. (2017). Regenerative intelligent brake control for electric motorcycles. Energies.

